# Clinical Severity in Different Waves of SARS-CoV-2 Infection in Sicily: A Model of Smith’s “Law of Declining Virulence” from Real-World Data

**DOI:** 10.3390/v15010125

**Published:** 2022-12-31

**Authors:** Emanuele Amodio, Dario Genovese, Alessandra Fallucca, Patrizia Ferro, Benedetta Sparacia, Luciano D’Azzo, Angelo Fertitta, Carmelo Massimo Maida, Francesco Vitale

**Affiliations:** Department of Health Promotion, Mother and Child Care, Internal Medicine and Medical Specialties “G. D’Alessandro”, University of Palermo, Via del Vespro 133, 90127 Palermo, Italy

**Keywords:** SARS-CoV-2, clinical outcomes, outcome assessment, health care, epidemiology, public health, COVID-19

## Abstract

Background: The COVID-19 epidemic had a rapid spread worldwide with a continuous and fast mutation of the virus, resulting in the emergence of several variants of concern (VOC). The aim of this study was to evaluate the severity of each VOC among SARS-CoV-2 infected subjects by investigating deaths, ICU admissions, intubations, and severe critical symptoms. Methods: An ecological observational study was performed to evaluate mortality rates and clinical characteristics of 321,490 unvaccinated Sicilian SARS-CoV-2 cases observed from 2 March 2020 to 27 March 2022. Odds ratios (OR) and 95% confidence intervals (CI) were calculated by multivariate logistic regression analysis evaluating factors determining a clinical worsening. Results: Delta (adj-OR 3.00, 95% Cls 2.70–3.33) and wild-type (adj-OR 2.41, 95% Cls 2.2–2.62) variants had a higher risk than the Omicron strain for developing critical COVID-19 necessitating intubation and eventually undergoing death. Moreover, males appeared to be significantly more susceptible to developing the worst clinical outcome considered, as did older subjects. Conclusions: The present study provides evidence of factors implicated in the worsening of SARS-CoV-2-infection-related clinical outcomes. The study highlighted the different roles of VOC, in particular Delta and wild-type, and being male and elderly in the development of a worse clinical outcome.

## 1. Introduction

The coronavirus disease 2019 (COVID-19) brought an unprecedented impact across the globe; the contagion initially emerged in Wuhan city, China, in December 2019, causing pneumonia-like symptoms in a cluster of patients [1]. The epidemic spread so rapidly around the world that the World Health Organization (WHO) declared the pandemic status a few months later [2]. In February 2020, Italy became the first European country to experience a SARS-CoV-2 outbreak [3]. Up to 31 March 2022, the overall number of confirmed infections was roughly 14,600,000, with differences reported between the various regions. Notably, at the end of March 2022, there were about 1 million cases observed in Sicily while, as of 5 December 2021, there were 340,146 confirmed cases, representing 6.8% of the resident population [4,5,6,7].

Despite the broad clinical spectrum, which ranges from an asymptomatic course to clinical manifestations of the respiratory tract requiring different types of treatment (domiciliary, hospital, intubation) [8], it is worth mentioning that in Italy, during the first phase of the COVID-19 pandemic, the high burden of SARS-CoV-2 infection led to a higher mortality rate and an increase in intensive care unit (ICU) demand due to severe-to-critical COVID-19 cases [9].

The development of vaccines, which resulted in a significant decrease in the severity of symptoms among infected people, revolutionized the history of the COVID-19 pandemic despite the existence of numerous clinical trials testing a therapy to inhibit viral entry or replication in the host [10,11,12,13,14].

The first COVID-19 vaccine, called BNT162b2, was approved for use in Italy on 23 December 2020 for the adult population (Comirnaty, Pfizer-BioNTech vaccine) [15]. Five months later, on 31 May 2021, the vaccination was extended to the pediatric population, and the vaccination coverage reached almost 90% in March 2022 for the two-dose complete cycle [16,17,18].

The virus is rapidly and continuously mutating, complicating the pandemic emergency. Like other RNA viruses, SARS-CoV-2 is prone to genetic evolution to adapt to human hosts, and adaptive mutations in the genome can alter the virus’s pathogenic potential [19]. Since its first appearance, numerous variants have been described; some of these have been classified by the WHO as variants of concern (VOCs) because of their increased transmissibility or virulence and, secondarily, because the antibodies produced from both natural infection and vaccination have lessened their neutralizing activity [20,21].

As of 31 May 2021, the WHO recommended the use of Greek alphabet letters to make public communications concerning variants clearer. Up to 31 December 2021, Italy faced the following VOCs: Alpha, Beta, Gamma, Delta, and Omicron [22].

In 1904, Theobald Smith, a bacteriologist and comparative pathologist, proposed the “law of declining virulence” based on his experimental work on Texas cattle fever. Smith’s theory was that, given the right conditions and sufficient time, pathogens should evolve to become less virulent over time [23,24]. This theory, although not applicable to infections in their entirety, appeared to be confirmed by mathematical models which predict that, once the endemic phase is reached, SARS-CoV-2 might be slightly virulent [25].

In order to test the Smith’s theory on the SARS-CoV-2 data, this study aimed to evaluate the severity of each VOC among SARS-CoV-2-infected subjects in terms of burden of disease by investigating the number of deaths, ICU admissions, intubations, and severe critical symptoms. To do so, the research group sought to explore which characteristics, including age, sex, and distinct VOCs, predicted a different COVID-19 outcome.

## 2. Materials and Methods

### 2.1. Data Sources

The present ecological observational study analyzes mortality rates and clinical characteristics of unvaccinated Sicilian COVID-19 patients from 2 March 2020 (10th week of 2020), which roughly corresponds to the first SARS-CoV-2 confirmed case in the Italian population, through 27 March 2022 (13th week of 2022).

Two electronic health registries, reporting the data collected by the Sicilian Regional Health Office under the supervision of the Italian Ministry of Health, were used to obtain data on participants who received COVID-19 vaccine (regional database “Vaccinazioni Anti-COVID-19”) and subjects who were infected with SARS-CoV-2 (regional database “Surveillance COVID-19 Platform”). Nasopharyngeal swabs for SARS-CoV-2 were performed in medical, domestic, and school settings. A PCR test was used to confirm COVID-19 cases.

The Italian identification code was used as a key to combine the two databases and assign the pertinent data from each database to the appropriate participant. The number of cases and the percentage distribution of VOCs by week within Italian territory were gathered from the European Centre for Disease Prevention and Control’s (ECDC) publicly available database [26].

The following information was extracted: demographics; SARS-CoV-2 infection time (expressed in weeks-years, WW-YYYY); the worst clinical symptoms experienced during the SARS-CoV-2 positivity; hospitalization; treatments received, including intubation; admission to intensive care unit (ICU); death; the number of positive cases per week; and the prevalence of each considered VOC per week: wild-type, Alpha, Beta, Gamma, and Omicron.

Regardless of the presence of signs and/or symptoms, subjects who tested positive for SARS-CoV-2 were referred to as “SARS-CoV-2-positive”.

According to the Italian National Classification Criteria in terms of COVID-19 severity [27], each infected individual who did not have symptoms was considered “asymptomatic”, whereas those with systemic general mild symptoms (e.g., fatigue, fever) but no clear evidence of respiratory tract infection, were referred to as “paucisymptomatic”. Since it was not possible to distinguish between “asymptomatic” and “paucisymptomatic” cases, these two categories were collapsed into “subclinical COVID-19” cases.

Moreover, we considered “mild COVID-19” each infected subject with any clinical manifestations of the respiratory tract/other organ systems that do not require hospital treatment; by converse, “severe COVID-19” cases were SARS-CoV-2-positive patients with clinical manifestations of the respiratory tract/other organs that require hospitalization. Finally, “intubated/deceased” cases were SARS-CoV-2-positive patients who were admitted to ICU, intubated, or who underwent death.

For the aims of this study, we excluded from the analysis subjects who:

- Received the first dose of a COVID-19 vaccine before contracting SARS-CoV-2.
- Developed SARS-CoV-2 infection in the weeks where the predominance of a VOC was less than 80%.

All the characteristics were retrieved from the Surveillance COVID-19 Platform since the COVID-19 vaccination database was utilized primarily to exclude vaccinated patients.

According to the previous criteria, a total of 321,490 SARS-CoV-2-positive subjects were analyzed.

### 2.2. Statistical Analysis

The normality distribution of continuous variables was assessed by the adjusted Jarque–Bera test (*n* > 5000) and, accordingly, all variables that were normally distributed were presented as means (standard deviation, SD), whereas non-normally distributed variables were summarized as median (interquartile range, IQR).

Categorical/discrete data were summarized by frequency and relative frequencies (%).

Odds ratios (OR) and 95% confidence intervals (CI) were calculated by a multivariate logistic regression analysis evaluating the factors that may determine the worsening of the clinical scenario in the context of SARS-CoV-2 infection. For this purpose, three different models were developed in order to evaluate the contribution of different independent variables (sex, age, cases per week, wild-type, Alpha, Beta, Gamma, Omicron) in determining the risk of developing mild COVID-19 or worse outcome (Model A), severe COVID-19 or worse outcome (Model B), or intubation/death outcome (Model C). In Model A, the referent was being subclinical cases; in Model B, the referent was having mild COVID-19 or being subclinical cases; in Model C, the referent was having developed mild-to-severe COVID-19 or being subclinical cases. Statistical significance was defined as *p* ≤ 0.05.

Analyses were performed using R Software analysis, version 4.0.5, R Foundation for Statistical Computing (Vienna, Austria) [28].

## 3. Results

### 3.1. Socio-Demographic and Clinical Characteristics

After the exclusion of vaccinated subjects and incomplete records, as reported in Table 1, a total of 321,490 Sicilian SARS-CoV-2 cases were included in the analysis, with a slightly higher prevalence of females (165,636; 51.5%). The median age was 32 years (IQR: 11–52 years). With regard to the worst clinical outcome, the great majority of the analyzed cohort contracted a subclinical SARS-CoV-2 infection (292,062; 90.9%), followed by mild COVID-19 cases (19,044; 5.9%).

### 3.2. Predominance of SARS-CoV-2 VOCs and Number of Positive Cases per Week

**Figure 1:**
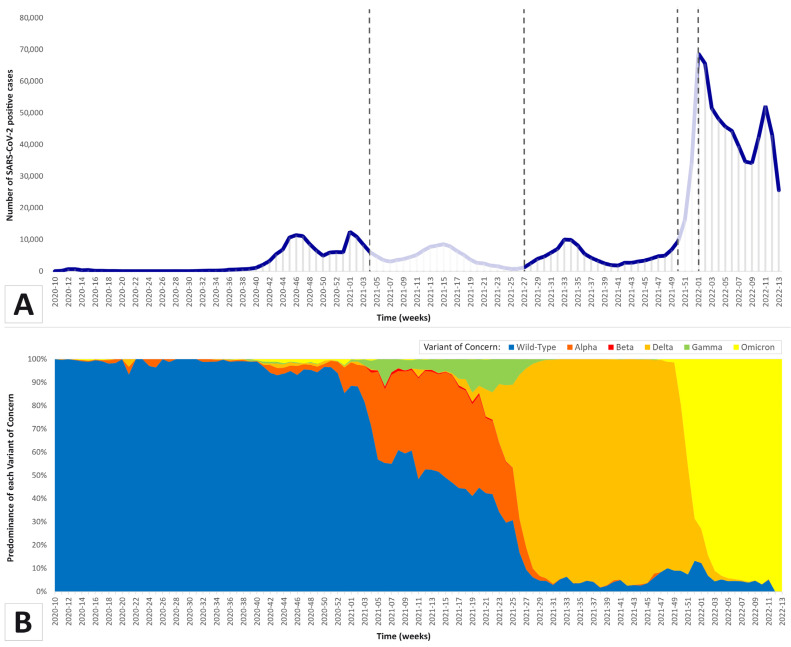
Distribution of SARS-CoV-2-positive cases each week from the 10th week of 2020 to the 13th week of 2022 (**A**) and the predominance of the five VOCs evaluated (wild-type, Alpha, Beta, Delta, Gamma, and Omicron) across the same weeks (**B**). The lighter parts of the curve, enclosed between the dashed lines, represent the cases that were excluded due to a predominance of one of the considered VOCs as less than 80%.

Figure 1 shows, in part A, the distribution of SARS-CoV-2-positive cases per week from the 10th week of 2020 to the 13th week of 2022. In parallel, in part B of the same figure, the predominance of the five VOCs evaluated (wild-type, Alpha, Beta, Delta, Gamma, and Omicron) is shown across the weeks. Some weeks of the epidemic curve were excluded from the analysis due to the predominance of one of the considered VOCs less than our cut-off value (80%).

The highest absolute frequencies of SARS-CoV-2-positive cases per week occurred in correspondence with the predominance of the Omicron strain with respect to the other VOCs (43,682.5 median cases per week during the Omicron-predominant period vs. 420 median cases per week and 4150.5 median cases per week during wild-type- and Delta-predominant periods, respectively). Furthermore, this Figure shows that Alpha, Beta, and Gamma variants were never predominant, and, consequently, they were excluded from the analyses since it was not possible to assess their involvement in the development of a worse clinical scenario derived from SARS-CoV-2 infection.

### 3.3. Multivariable Analyses

**Figure 2:**
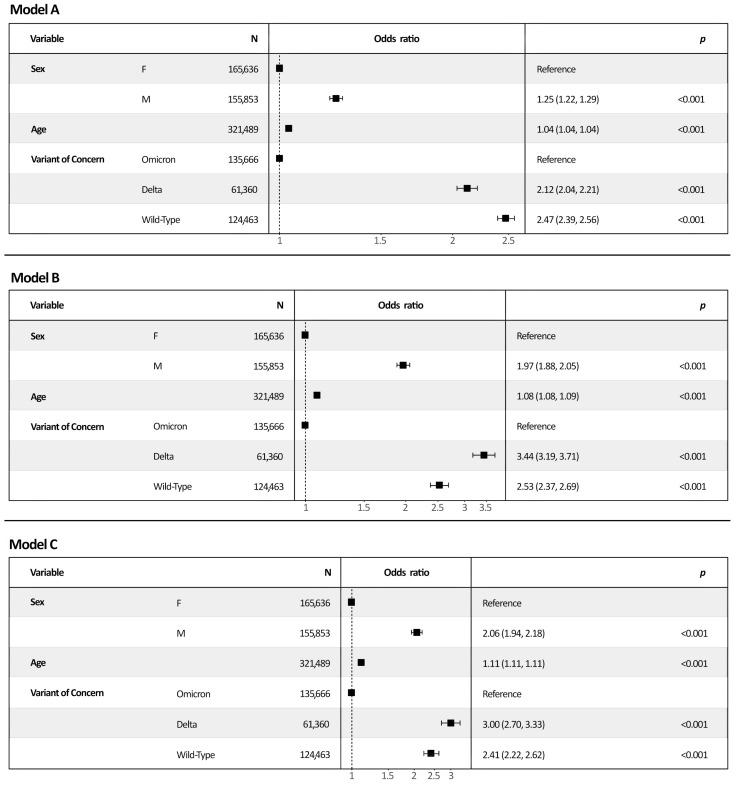
Visual representation of multivariable logistic regression models and odds ratios (95% CI). **Legend**: Model A included as outcome mild COVID-19 or worse (referent was being subclinical cases); Model B included as outcome severe COVID-19 or worse (referent was having mild COVID-19 or being subclinical cases), and Model C included as outcome intubation/death (referent was having developed mild-to-severe COVID-19 or being subclinical cases).

In Figure 2, three different multivariable logistic regression analysis models are reported with different outcomes: mild COVID-19 or worse (Model A); severe COVID-19 or worse (Model B); and intubation/death (Model C).

For each model, males appeared to be significantly more susceptible to developing the worst clinical outcome considered. Moreover, the odds of developing the worst clinical outcome showed a statistically significant increase in risk per year of age (4% against mild COVID-19 or worse, 8% against severe COVID-19 or worse, and 11% against intubation/death). Regarding the VOCs, taking as referent the Omicron strain, it was shown that the odds of developing the worst clinical outcome considered in each different model are statistically significantly higher in subjects who were likely infected with Delta or wild-type strains.

Model A suggested that, when compared to Omicron, both Delta (adj-OR 2.12, 95% Cls 2.04–2.21, *p*-value <0.001) and wild-type (adj-OR 2.47, 95% Cls 2.39–2.56, *p*-value <0.001) variants more probably cause infections which may develop a mild-to-severe COVID-19 outcome. Similarly, Model B showed that Delta (adj-OR 3.44, 95% Cls 3.19–3.71, *p*-value <0.001) and wild-type (adj-OR 2.53, 95% 2.37–2.69, *p*-value <0.001) infections are more likely than Omicron-related infections to undergo a severe COVID-19 outcome. Finally, Model C also assessed that Delta (adj-OR 3.00, 95% Cls 2.70–3.33, *p*-value <0.001) and wild-type (adj-OR 2.41, 95% Cls 2.2–2.62, *p*-value <0.001) variants had a higher risk than the Omicron strain to develop such a critical COVID-19 as to necessitate intubation and eventually undergo death.

## 4. Discussion

The subsequent emergence of SARS-CoV-2 variants has caused multiple waves of COVID-19 outbreaks around the world. Numerous distinct viral strains have spread since the WHO declared the pandemic in March 2020 [29], and an investigation of epidemiological characteristics of the different VOCs might be useful to comprehend the severity of this pandemic and guide current and future public health strategies.

An interesting finding of the present study is the higher incidence of the Omicron strain compared to the other variants. It has been shown that during the Omicron-predominant period, the number of cases was on median higher than during the wild-type- and Delta-predominant periods. Although the spread of the different VOCs could be related to the control strategies (lockdown, surgical mask use, etc.), the increased transmissibility of Omicron is supported by several studies that estimated that the Omicron variant can infect 3 to 6 times more people than the Delta strain [30,31,32]. However, transmissibility should be considered in relation to disease severity.

In fact, the best management of COVID-19 patients in both domiciliary and hospital settings may be achieved by assessing the factors responsible for the worsening of the clinical outcome among SARS-CoV-2-positive patients. In our study, to answer this question, we performed three different multivariable regression models.

Each of the three multivariable regression models showed that male sex and the unitary increment per year of age increase the risk of death and intubation. These results seem to be confirmed by other studies. According to Mohiuddin and Kasahara [33], cellular senescence can aggravate the clinical pattern of patients with COVID-19, and this is prominent in elderly patients [34]. As shown in a recent study carried out in England, men are 41% more likely than women to be admitted to the hospital, but they also have a 62% greater chance of death [35]. Furthermore, a meta-analysis implemented by Peckham et al. [36] found that while males and females both have an equal risk of infection, men are more likely to develop severe disease. The study identified the male sex as a risk factor for death and intensive care admissions, which is consistent with our findings.

The main aim of this study was to evaluate the impact of each VOC among the unvaccinated individuals, trying to assess whether virulence, incidence, and fatality rate would differ between the different VOCs. Multivariable regression models allowed us to assess which VOCs worsen the clinical scenario of SARS-CoV-2-positive patients. Unfortunately, we could not determine with certainty which of the variants was responsible for each positive subject; nevertheless, we decided to attribute to each record the VOC whose predominance during the week of detection was at least 80%. Overall, our findings may suggest a decreased virulence of Omicron in comparison to wild-type and Delta when severe outcomes, intubation, and death are considered. Nevertheless, as Theobald Smith postulated in his “law of declining virulence”, the lower severity of the Omicron variant may also be attributed to the natural protection inherited with previous immunity [37]. Accordingly, in an early assessment of the clinical severity of the Omicron strain in South Africa, Wolter et al. [38] observed that, after adjusting for characteristics associated with disease severity, patients who previously had a Delta variant infection, when infected with Omicron, had a markedly lower chance of severe complications (62.5% vs. 23.4%; adj-OR: 0.3, 95% Cis: 0.2–0.5). In accordance with the prior research, the retrospective study carried out by Nordström et al. [39] on the entire Swedish population revealed the existence of effective natural immunity in safeguarding against hospitalization after up to 20 months. Indeed, for the follow-up time considered, natural immunity was linked with a 95% reduced risk of SARS-CoV-2 infection (adj-HR: 0.05, 95% CIs: 0.05–0.05; *p* < 0.001) and an 87% lower risk of COVID-19 hospitalization (adj-HR: 0.13, 95% CIs: 0.11–0.16; *p* < 0.001). However, it should be noted that less than 7% of the population in Sicily at the time of the Omicron spread (December 2021) had a prior infection. As a result, natural immunity to SARS-CoV-2 prior infection may have had a marginal impact on the reduction in severity of the Omicron strain.

There is a dearth of scientific literature regarding the clinical severity amongst unvaccinated subjects. However, a US observational study by Lauring et al. [40] revealed that in unvaccinated cases (*n* = 3959), the severity of COVID-19 was highest for the Delta variant group (adj-OR 1.28, 95% CIs 1.11–1.46) when taking as referent the Alpha strain; at the same time, taking as referent the Delta variant, the severity of COVID-19 was lowest for the Omicron strain group (adj-OR 0.61, 95% CIs 0.49–0.77). Similar results were observed by Wang et al. [41], who conducted a cohort study in the United States involving 651,640 children aged 5 or younger between 1 September 2021 and 31 January 2022. Their findings suggested that the incidence rate during Omicron-predominant period was 6 to 8 times that of the Delta-predominant period, while severe clinical outcomes were less common than with the Delta strain.

The present study encountered some limitations. First, it was not possible to determine with certainty which of the VOCs was responsible for each positive subject. Second, data about comorbidities, lifestyle risk factors, or behaviors were not available, and, therefore, it was not possible to stratify our cohort for pre-existing diseases, immunodeficiency disorders, immunosuppressive therapies, smoking habits, and alcohol use, and, at the same time, it was not possible to quantify time of exposure deriving from the recommendations for the prevention of COVID-19 (hand hygiene practices, wearing mask habits, and usage time, etc.). Third, the registration of clinical severity suffered from the operator’s discretion and the patients’ self-declaration, leading to a potential misclassification which is not quantifiable, especially in regard to mild COVID-19 cases. Finally, it could not be excluded that Omicron was characterized by lower virulence because of a better clinical and therapeutic approach due to the increased knowledge over time.

## 5. Conclusions

The present study provided evidence of factors implicated in the worsening of SARS-CoV-2-infection-related clinical outcomes. The study highlighted the different roles of each VOC and supports the fact that being male and elderly predisposes patients to the development of a worse clinical outcome. Overall, from wild-type to Omicron variants, a decrease in viral virulence has been evident, in agreement with Smith’s theory; notwithstanding, the role of natural immunity must be acknowledged as well.

The present study’s findings should be considered when discussing health and social policies, given the consistent dataset size and the analyzed period (from the beginning of the pandemic to the 13th week of 2022). Further studies should be conducted to estimate newer SARS-CoV-2-variant-related burdens of disease.

## Figures and Tables

**Table 1:** Socio-demographic and clinical characteristics of the COVID-19 cases included in the study.

	Unvaccinated Population (*n* = 321,490)
**Sex, *n* (%)** - *F* - *M*	165,636 (51.5%) 155,854 (48.5%)
**Age, Median (IQR)**	32 (11–52)
**Worst clinical outcome, *n* (%)** - *Subclinical SARS-CoV-2 infection* - *Mild COVID-19* - *Severe COVID-19* - *Intubation/Death*	292,062 (90.9%) 19,044 (5.9%) 4502 (1.4%) 5882 (1.8%)

## Data Availability

Data will be made available upon reasonable request to the corresponding author.
